# Biomechanical evaluation of a biomimetic spinal construct

**DOI:** 10.1186/s40634-014-0003-z

**Published:** 2014-06-26

**Authors:** Tian Wang, Jonathon R Ball, Mattew H Pelletier, William R Walsh

**Affiliations:** Surgical & Orthopaedic Research Laboratories, Prince of Wales Clinical School, University of New South Wales, Clinical Science Bldg, Prince of Wales Hospital, Gate 6, Avoca Street, 2031 Sydney, Australia; Graduate School of Biomedical Engineering, University of New South Wales, Sydney, NSW Australia; Royal North Shore Hospital, St Leonards, NSW Australia

**Keywords:** Synthetic, Lumbar, Sowbones, Biomechanical, Interbody cage, Lateral plate, Anterior plate, Pure moment, Sawbones, Motion segment, Fatigue

## Abstract

**Background:**

Laboratory spinal biomechanical tests using human cadaveric or animal spines have limitations in terms of disease transmission, high sample variability, decay and fatigue during extended testing protocols. Therefore, a synthetic biomimetic spine model may be an acceptable substitute. The goal of current study is to evaluate the properties of a synthetic biomimetic spine model; also to assess the mechanical performance of lateral plating following lateral interbody fusion.

**Methods:**

Three L3/4 synthetic spinal motion segments were examined using a validated pure moment testing system. Moments (±7.5 Nm) were applied in flexion-extension (FE), lateral bending (LB) and axial rotation (AR) at 1Hz for total 10000 cycles in MTS Bionix. An additional test was performed 12 hours after 10000 cycles. A ±10 Nm cycle was also performed to allow provide comparison to the literature. For implantation evaluation, each model was tested in the 4 following conditions: 1) intact, 2) lateral cage alone, 3) lateral cage and plate 4) anterior cage and plate. Results were analysed using ANOVA with post-hoc Tukey’s HSD test.

**Results:**

Range of motion (ROM) exhibited logarithmic growth with cycle number (increases of 16%, 37.5% and 24.3% in AR, FE and LB respectively). No signification difference (p > 0.1) was detected between 4 cycles, 10000 cycles and 12 hour rest stages. All measured parameters were comparable to that of reported cadaveric values. The ROM for a lateral cage and plate construct was not significantly different to the anterior lumbar interbody construct for FE (p = 1.00), LB (p = 0.995) and AR (p = 0.837).

**Conclusions:**

Based on anatomical and biomechanical similarities, the synthetic spine tested here provides a reasonable model to represent the human lumbar spine. Repeated testing did not dramatically alter biomechanics which may allow non-destructive testing between many different procedures and devices without the worry of carry over effects. Small intra-specimen variability and lack of biohazard makes this an attractive alternative for in vitro spine biomechanical testing. It also proved an acceptable surrogate for biomechanical testing, confirming that a lateral lumbar interbody cage and plate construct reduces ROM to a similar degree as anterior lumbar interbody cage and plate constructs.

## Background

Development of synthetic replicates has been driven by the challenges associated with using fresh tissues for biomechanical testing. Beyond scarcity and cost considerations, fresh samples are biohazards and require appropriate personal protective equipment as well as the enactment of handling and specimen tracking procedures. There are also social and ethical concerns that must be addressed. Additional limitations that are particularly relevant to the spine include fluctuations in soft and hard tissues properties over time and with exposure to air, which alters the kinematic response of motion segments [1]. During high cycle fatigue tests spinal segments are likely to suffer from tissue putrefaction. Freezing tissues offers an means to hinder decomposition, however initial freezing itself will also influence mechanical properties [2]. With the amount of time between expiration of the donor and subsequent freezing uncontrolled variables, this is likely to further influence the condition of the tissues. Initial tissue quality is also an issue as the majority of the cadaveric specimen is from elderly donors with osteoporosis, or other degenerative pathologies. Inherent inter-specimen variability in size, bone quality and disc pressure [3] will increase data scatter. Animals are a suitable replacement for human cadaveric spines in certain testing protocols [4–11] with well reported anatomical and biomechanical comparisons [12,13] but have limitation in terms of size and angle of lordosis. While these samples may be more easily accessible and more cost effective, they still embody the previously mentioned limitations with human tissues.

The creation of a biomechanically accurate joint complex including soft tissues presents a challenge beyond that of modeling individual bones [14–18]. Further complicating this process in the spine is that intervertebral joints consist two articulating synovial joints (zygapophyseal joints) and one symphysis (intervertebral disc), as well as surrounding syndesmoses (anterior and posterior longitudinal, inter- and supraspinous, intertransverse ligaments and ligamentaflava).

A novel synthetic spinal testing models recently released by Sawbones may have utility in research and development. The current study examined the anatomy and biomechanical aspects of this synthetic biomimetic spine model in cyclic testing; and to evaluate anterior and lateral plating with interbody cages, to address the following questions: 1) is a lateral interbody cage + plate construct mechanically comparable to an anterior interbody cage + plate construct; and 2) is a stand-alone lateral interbody cage construct mechanically comparable to the intact state.

## Methods

### Evaluation of anatomy

Three L3/4 synthetic spinal motion segments, recently developed by SawBones (Vashon, WA, USA) were evaluated. Samples were examined using clinical computed tomography (CT Aquilion PRIME; Toshiba, Tokyo, Japan) with 0.5 mm slices. 3D models, which were built with Mimics (Materialise, Leuven, Belgium) from the DICOM data, were used to measured anatomy parameters such as end plate depth, end plate width, vertebral body height, spinal canal depth, spinal canal width, pedicle height and pedicle width. Results were compared with anatomical data from the human [19] and sheep [12] lumbar spine.

### Cyclic tests

Motion segments were examined with a validated pure moment testing system [20,21]. Moments (±7.5 Nm) were applied in flexion-extension (FE), lateral bending (LB) and axial rotation (AR) at 1Hz for total 10000 cycles in MTS Bionix (MTS Systems, Eden Prairie, MN) with 25 KN axial-torsional load transducer (model number: 662.20D-05, MTS system corporation, Eden Prairie, MN, USA). Motion was assessed at 4, 125, 250, 500, 1000, 2500, 5000 and 10000 cycles. A non-contacting thermometer was used to measure the temperature of disc, endplate and vertebral body at each assessment stage. After a 12 hour rest period, an additional test was run at ±7.5 Nm to determine the influence of temperature (designed as recovery group test). A ±10 Nm cycle was performed to allow additional comparison to the literature, and act as the intact group (INT) for subsequent instrumented tests. A near infrared 3D motion tracking system with retro-reflective markers (Osprey, Motion Analysis, Santa Rosa, CA) was employed to record the motion of the sample. Pretesting calibration found the system accuracy within 0.1 mm in displacement and 0.1° in rotation. Post processing was performed with an in-house written script MATLAB (MathWorks, Natick, MA) to extract the torque-angle curves and resulting range of motion (ROM) and neutral zone (NZ). NZ was calculated based on the Wilke et al. [22].

### Implanted conditions

**Figure 1:**
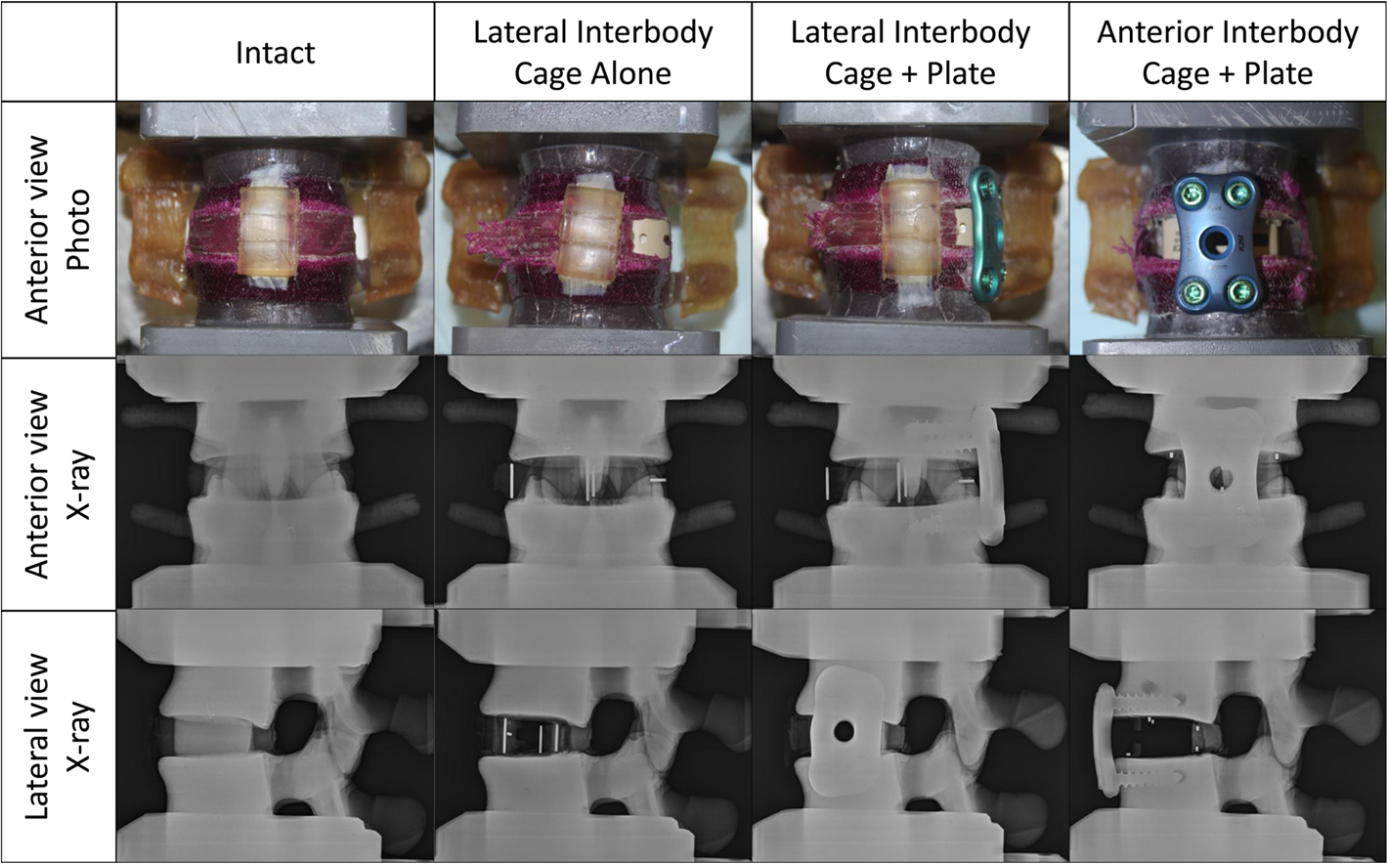
**Anterior view photo, anterior view x-ray and lateral view x-ray imaging of different sample conditions.**

Four cycles (±10 Nm) were applied in the primary anatomical planes. The intact group (INT) results were obtained during the cycle test mentioned above. Each sample was tested in three additional states (Figure 1), 1) lateral interbody cage alone (LIC); 2) lateral interbody cage + plate (LICP); and 3) anterior interbody cage + plate (AICP). PEEK interbody cages (K2M Aleutian, Life Health Care, North Ryde, Australia) were used in this study. The rigid plating system used consisted of a locking plate and four cancellous screws (K2M Cayman, LifeHealthCare, North Ryde, Australia). The implants were sized appropriate to specimen anatomy. Firstly, a box-like incision was made through the lateral annulus followed by discectomy. The disc material was removed using a surgical curette and rongeur. The anterior longitudinal ligament and part of anterior annulus was still connected at this stage. The lateral cage (14 mm × 8°) was implanted, and mechanically tested (LIC). A43 mm lateral locking plate was secured with four 6.0/28 mm cancellous screws and mechanically tested (LICP). The lateral interbody cage and plate was removed, the anterior longitudinal ligament and annulus were cut through and a complete discectomy performed. The anterior interbody cage (15 mm × 10°) and plate (38 mm with 6.0/28 mm screws) were then applied and the sample tested (ALCP). Radiographs were taken before and after testing with a high resolution X-ray (MX-20; Faxitron, Tucson, AZ).

### Statistical analysis

Data from cyclic tests was analyzed using one way ANOVA with a 0.05 significance level with IBM SPSS Statistics (version 21, IBM, Armonk, NY), followed by a Tukey HSD post hoc analysis. For implanted conditions, ANOVA was again applied followed by Tukey HSD post hoc analysis between test conditions.

## Results

### Anatomy

L3 and L4 anatomical parameters of the synthetic spine model are compared with published human [19] and sheep [12] L3 and L4 data in Table 1. The synthetic model anatomy showed a close match to the human, while the sheep differs to the other two groups, as expected.

**Table 1:** **Anatomical comparison between synthetic model, human cadaver and sheep; (l) indicates inferior or caudal level; number in brackets indicated the standard deviation**

	**L3**			**L4**		
	**Synthetic model**	**Human**	**Sheep**	**Synthetic model**	**Human**	**Sheep**
End plate depth(l)	35.48 (0.46)	34.80 (1.24)	20.00 (0.60)	37.09 (1.05)	33.90 (0.85)	20.10 (0.70)
End plate width(l)	49.85 (0.76)	48.00 (1.24)	29.80 (1.30)	52.36 (0.61)	49.50 (1.38)	31.00 (0.60)
Vertebral body height	26.76 (0.03)	23.80 (1.10)	40.20 (1.20)	23.96 (0.86)	24.10 (1.10)	41.10 (0.80)
Spinal canal depth	20.62 (0.19)	17.50 (0.53)	8.60 (0.70)	21.38 (0.45)	18.60 (0.71)	8.80 (0.40)
Spinal canal width	23.53 (0.80)	24.30 (0.64)	12.60 (1.10)	23.37 (1.05)	25.40 (0.49)	12.90 (1.10)
Pedicle height	13.93 (0.90)	14.40 (0.62)	35.70 (1.40)	16.49 (0.44)	15.40 (0.46)	36.30 (1.40)
Pedicle width	12.88 (0.85)	10.20 (0.60)	9.50 (0.90)	11.49 (0.27)	14.10 (0.46)	9.60 (1.00)

### Cyclic tests

**Figure 2:**
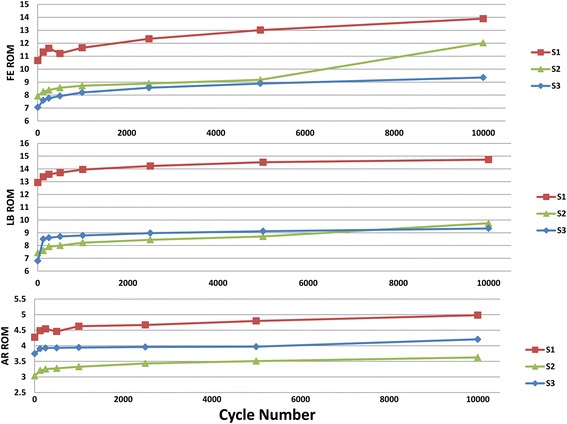
**Individual sample ROM results against cycle number.** Note the overall logarithm change occurring in relation to cycle number. S1-Sample 1, S2-Sample 2, S3-Sample 3; angle is represented on the Y-axis. FE-flexion/extension; LB-lateral bending; AR-axial rotation.

**Figure 3:**
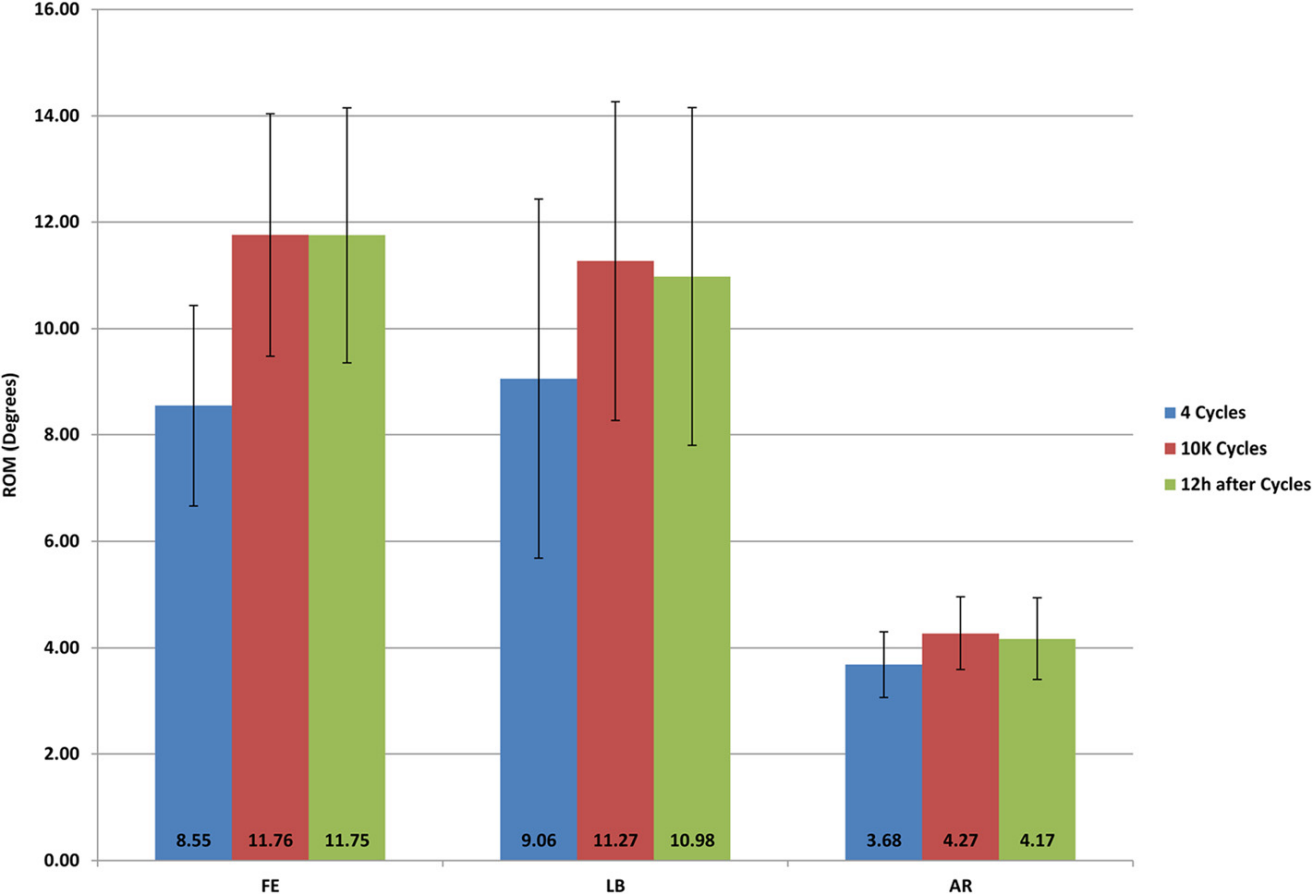
**ROM comparison between 4 cycles, 10 K cycles and recovery.** FE-flexion/extension; LB-lateral bending; AR-axial rotation.

Figure 2 shows the individual sample FE, LB and AR ROM results against cycle number. After 10 K cycles, the average ROM of three samples increased 16%, 37.5% and 24.3% in AR, FE and LB respectively. All samples displayed logarithmic growth in ROM with increasing cycle number. However, it was also noted that the absolute value of the ROM differed for sample1against other two, particularly in LB. The mean value for ROM increased from 4 to 10 K cycles and decreased slightly following the recovery period (Figure 3); however no signification differences (p > 0.1) were detected between any of these groups. When compared with the 10 Nm cadaveric test results of Panjabi et al. [23] and [24] (Table 2), synthetic ROM were similar to the human data. And for NZ results, most FE NZ and LB NZ were in the range of the cadaveric data, but the AR NZ was smaller than reported human results. Furthermore, compared with human data, the synthetic spine reduced standard deviation by 10%, 30% and 57.5% in AR, FE, and LB ROM respectively.

**Figure 4:**
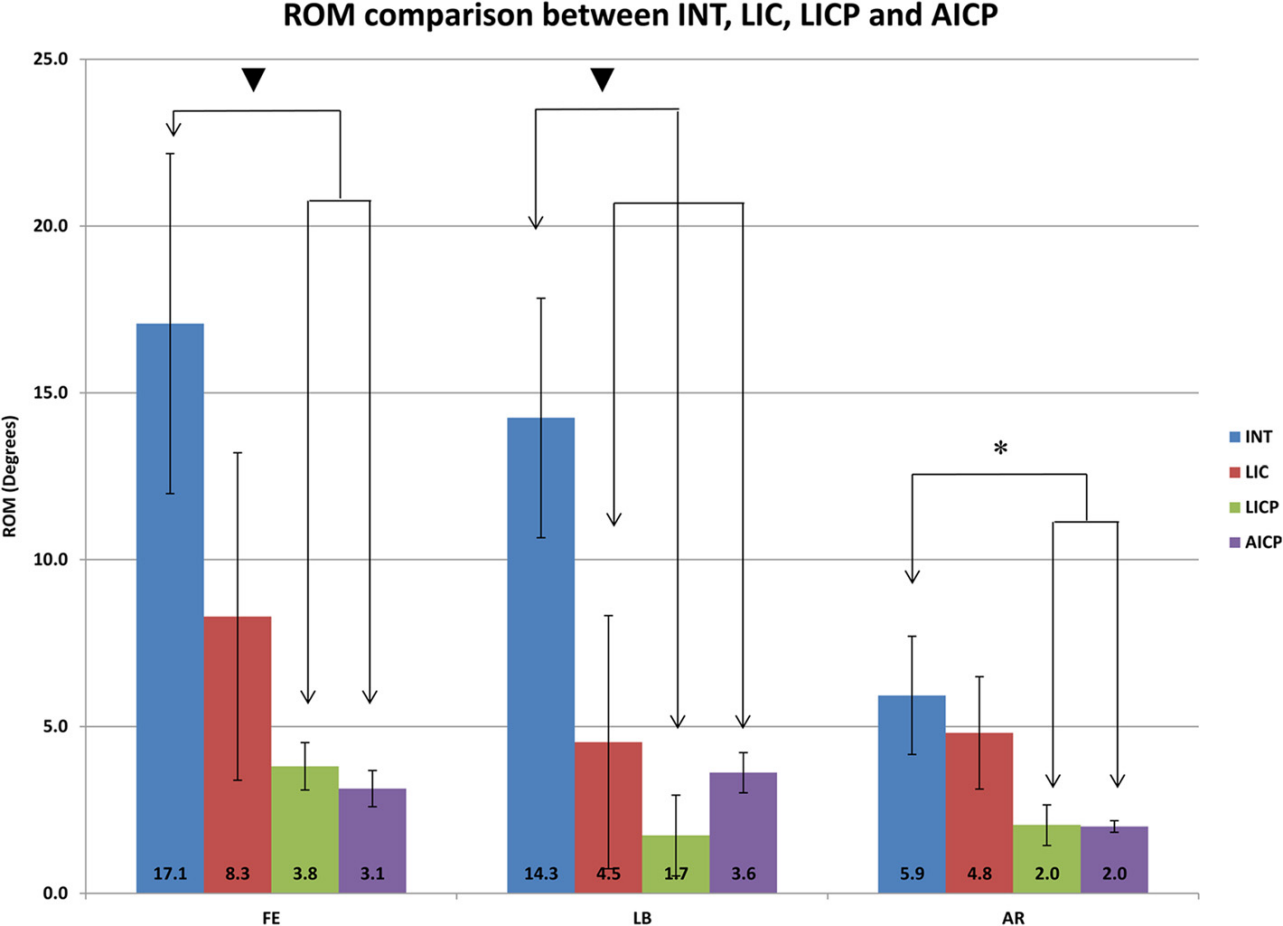
**ROM comparison between intact (INT), lateral interbody cage alone (LIC), lateral interbody cage + plate (LICP), and anterior interbody cage + plate (AICP) group.** ▼ = p < 0.01; * = p < 0.05.FE-flexion/extension; LB-lateral bending; AR-axial rotation.

**Table 2:** **10 Nm ROM and NZ results comparing with human cadaveric data**

	**ROM (degree)**				**NZ (degree)**			
	**s1**	**s2**	**s3**	White [24]	**s1**	**s2**	**s3**	Panjabi [23]
FE	22.9	15.7	12.8	15.0 (7.0)	0.8	0.9	0.9	0.7 (0.6)
LB	18.4	12.7	11.7	16.0 (8.0)	2.3	1.1	1.0	0.9 (0.8)
AR	8.0	5.1	4.8	5.2 (2.0)	0.8	0.8	0.7	1.3 (0.4)

S1-Sample 1, S2-Sample 2, S3-Sample, number in brackets indicated the standard deviation.

### Implanted conditions

As expected, the ROM of the lateral cage alone group was less than the intact group, and was further reduced with the plate fixation (Figure 4). Quantitative results shown that the ROM of flexion/extension decreased by 51.5% with a lateral cage alone. Further decrease was obtained with a lateral cage + lateral plate (77.8%); this magnitude of reduction was similar to that seen with an anterior cage + plate (81.7%). ROM in lateral bending decreased by 68.2% with a lateral cage alone and further decreased with a lateral cage + plate (87.9%). This magnitude of reduction was greater than that seen with an anterior cage + plate (74.7%). Axial rotation ROM decreased minimally with a lateral cage alone (18.9%), and to a greater extent with a lateral cage + plate (65.6%). This magnitude of reduction was similar to that seen with an anterior cage + plate (66.3%). In addition, cadaveric studies which investigate ROM of lateral interbody cage fusion with and without plate fixation [25–28] (Table 3) correlate well with the current study. Statistical analyses revealed differences between the intact and two cage + plate groups (p < 0.05 in AR and p < 0.01 in FE and LB). No statistic differences were detected between lateral cage + plate and arterial cage + plate; or between intact and lateral cage alone groups, except in LB (p < 0.01).

**Table 3:** **Comparison of the current study results with relevant studies**

**Author/Publication**	**Applied torque [N · m]**	**Test conditions**	**FE ROM [% Intact]**	**LB ROM [% Intact]**	**AR ROM [% Intact]**
Current study	±10	Lateral cage alone	48.5%	31.4%	81.3%
		Lateral cage and plate	22.2%	11.9%	33.9%
Cappuccino et al., 2010 [26]	±7.5	XLIF cage alone	31.6%	32.5%	69.4%
		XLIF + lateral plate	32.5%	15.9%	53.4%
Bess et al., 2008 [25]	±5	XLIF cage alone	45.8%	41.8%	66.3%
		XLIF + lateral plate	40.0%	24.2%	50.7%
Le Huec et al., 2002 [28]	±7	LLIF cage alone	71.3%	88.5%	107.7%
		LLIF cage + lateral plate	40.3%	27.3%	45.2%
Kim et al., 2005 [27]	±7.5	LLIF graft alone	75.2%	96.9%	71.6%
		LLIF + lateral plate	52.1%	37.9%	41.3%

XLIF-eXtreme Lateral Interbody Fusion; LLIF – Lateral Lumbar Interbody Fusion.

## Discussion

The present study evaluated the response of the novel synthetic spine models to pure moment loading in all three anatomical planes under high cycle testing as well as following implantation with interbody cages and anterior/lateral plates. The anatomy of the synthetic spine compared to published data [12,19] was also reported. Results showed this synthetic spine model presents a better simulation in terms of anatomy than other spine surrogates for the human spine [29–36] (Table 1). The intact synthetic model has comparable biomechanics compared to published results for intact human cadaveric spine segments [23,24] (Table 2). A key measure of spinal kinematics is ROM. When comparing with human spines, ROM results were within the range of the human data, except AR ROM for sample1. NZ represents the lax portion of bending where relatively small loads cause large rotations [23]. Overall, NZ for FE and LB were within the human data range. In addition, the smaller standard deviation in ROM for all modes of bending, when compared with human data, would be expected to increase repeatability when used for this type of scientific research.

The 10,000 cycle test sequence results represented a long test life of this synthetic model. Although this would not be expected to represent true fatigue testing of implanted constructs in a spine, it is likely to span the working life of the spine in a laboratory environment. Results showed that ROM logarithmically increased with cycle number (Figure 2). Although after 10 K cycles ROM in all three motions had different magnitudes of increase, the final round of tests showed that the ROM was still within range of human.

The decrease in stiffness is likely due to two primary reasons: material property changes and mechanical bonding failure brought by fatigue. Material properties may change permanently due to microscopic failure points induced by cyclic loading, or transiently due to local temperature change. Traditional measures of spinal biomechanics present a torque/angle plot containing a hysteresis curve. This hysteresis area represents the amount of energy that is lost during the cycle. This energy is expended by causing permanent damage or is lost as heat. In the present test, a non-contacting thermometer showed that after 10 K cycles, local temperature increased up to 5°C in FE and LB motions at the intersection between disc and vertebral body. An additional source of heat may have been friction between the different material components. However, the ROM did not change significantly following cooling and recovery over 12 hours (Figure 3). This suggests that the temperature increase may not be the primary driver for changing mechanical properties. Visual inspection found that there were fibers broken in the outer annulus fibrosis, particularly at the intersection between disc and vertebral body. This area represents an abrupt change in material properties which the simulated outer annulus helped to transition. Failure of this construct is likely to have been responsible for a fair portion of the changing biomechanics.

Repeated testing with differing implanted conditions showed the influence of interbody cage placement and plating on spinal biomechanics (Figure 4). Compared with the intact state, lateral interbody cage placement did not affect AR, trending towards a significant reduction in FE (p = 0.06), and significantly reduced in LB. The geometry and size of the lateral cage provides support in LB via a large footprint and extended lateral dimension, which likely contributed to these findings.

Differences in testing methodology make it difficult to draw exact comparisons with other cadaveric biomechanical studies. However, similar trends in outcomes can be identified [25] and [26] used a human cadaver model to compare lumbar spine kinematics of a laterally placed interbody device used as a stand-alone construct with various instrumented constructs (Table 3). They tested 5 conditions: (1) intact spine, (2) lateral discectomy and stand-alone lateral interbody device (XLIF), (3) XLIF supplemented by a lateral plate, (4) XLIF supplemented by unilateral pedicle screws, and (5) XLIF supplemented by bilateral pedicle screws. Results revealed that the extreme lateral interbody implant, with or without supplemental fixation, provides primary stabilization in all loading modes compared with intact specimens. The greatest reduction in ROM was observed with lateral bending and flexion-extension in all treatments. Le Huec et al. [28] compared the biomechanics of lateral interbody cage in 8 cadaveric lumbar functional spinal units with 2 additional modes of fixation: a lateral plate and a lateral plate locked to the cage. The laterally placed cage produced a decrease in the ROM compared with intact in flexion extension and lateral bending, but not in axial rotation. The reduction in ROM observed with the plate was significantly reduced relative to the intact spine in all 3 motion planes. Kim et al. [27] evaluated the stability of human cadaveric lumbar spine constructs with interbody reconstruction performed via a traditional ALIF approach or a lateral approach. Specimens were evaluated in 4 conditions: (1) intact spine, (2) destabilization by anterior or lateral discectomy, (3) stand-alone interbody reconstruction and (4) interbody reconstruction supplemented with additional fixation. The stand-alone lateral interbody and ALIF implants restored the ROM and NZ to intact spine values. Compared with the intact spine, supplemental instrumentation significantly reduced the ROM and NZ in all loading modes.

In summary, these cadaveric tests results indicated three primary points. Firstly, the stand-alone lateral interbody cage provided primary stabilization, which is supported by the present study. The present study showed that lateral interbody cage alone can reduce the ROM 48.5% and 31.4% in flexion-extension and lateral bending respectively. Similar reduction can be found in published literature (Table 3). Second, a stand-alone cage did not provide sufficient axial rotational stabilization (66.3%-107.7%), and this was confirmed again by the present study (81.3%). Third, a supplementary lateral plate with screws significantly reinforces the stabilization in all directions. Again, this is supported by the current study. However, a larger reduction in ROM has been found in current study compared to cadaveric data (22.2%, 11.9% and 33.9% in FE, LB and AR respectively vs. 32.5-52.1%, 15.9-37.9%, and 41.3-53.4% with cadaveric sample). This might due to the different loads applied. It also should be noted that the vertebral bodies do not contain simulated cancellous bone. The solid nature of the vertebrae may overestimate the fixation achievable by screws and limit the amount of deformation at the disc/endplate interface.

### Limitations

The primary limitation of this study is the small sample size which did not allow robust statistical analysis. Additionally no cadaveric samples were tested for direct comparison. To allow comparison with cadaveric values published results were utilized. This provided mean values but did not allow for any statistical analysis. The destructive nature of the implanted conditions dictated that each sample follow a standard order. Randomized tests with individual samples for each intact and implanted condition may be desirable for future testing.

## Conclusion

This study found that based on the anatomy and biomechanical similarities, the synthetic spine tested here provides a reasonable model to represent the human lumbar spine. Repeated testing did not dramatically alter biomechanics which may allow non-destructive testing between many different procedures and devices without the worry of carry over effects. Small intra-specimen variability and lack of biohazard makes this an attractive alternative for in vitro spine biomechanical testing. However, a preconditioning test should be run to check the initial ROM of each sample. It also proved an acceptable surrogate for biomechanical testing, confirming that a lateral lumbar interbody cage and plate construct reduces ROM to a similar degree as anterior lumbar interbody cage and plate constructs. Clearly, more testing is required in the future to evaluate this synthetic model for more complex applications.

## Abbreviations

ROM: Range of motion
NZ: Neutral zone
FE: Flexion-extension
LB: Lateral bending
AR: Axial rotation
INT: Intact group
LIC: Lateral interbody cage alone
LICP: Lateral interbody cage with plate
AICP: anterior interbody cage with plate
XLIF: Lateral discectomy and stand-alone lateral interbody device
LLIF: Lateral lumbar interbody fusion
ALIF: Anterior lumbar interbody fusion
